# A Proposed Geobiology-Driven Nomenclature for Astrobiological *In Situ* Observations and Sample Analyses

**DOI:** 10.1089/ast.2020.2318

**Published:** 2021-08-16

**Authors:** Scott M. Perl, Aaron J. Celestian, Charles S. Cockell, Frank A. Corsetti, Laura M. Barge, David Bottjer, Justin Filiberto, Bonnie K. Baxter, Isik Kanik, Sally Potter-McIntyre, Jessica M. Weber, Laura E. Rodriguez, Mohit Melwani Daswani

**Affiliations:** ^1^ NASA Jet Propulsion Laboratory, California Institute of Technology, Pasadena, California, USA.; ^2^ Mineral Sciences, Natural History Museum of Los Angeles County, Los Angeles, California, USA.; ^3^ Blue Marble Space Institute for Science, Seattle, Washington, USA.; ^4^ School of Physics and Astronomy, University of Edinburgh, Edinburgh, Scotland.; ^5^ Department of Earth Sciences, University of Southern California, Los Angeles, California, USA.; ^6^ Lunar and Planetary Institute, Houston, Texas, USA.; ^7^ Great Salt Lake Institute, Westminster College, Salt Lake City, Utah, USA.; ^8^ School of Earth Systems and Sustainability, Southern Illinois University Carbondale, Carbondale, Illinois, USA.

**Keywords:** Biogenicity, Nomenclature, Geobiology, Mars Sample Return, Evaporites. Astrobiology 21, 954–967

## Abstract

As the exploration of Mars and other worlds for signs of life has increased, the need for a common nomenclature and consensus has become significantly important for proper identification of nonterrestrial/non-Earth biology, biogenic structures, and chemical processes generated from biological processes. The fact that Earth is our single data point for all life, diversity, and evolution means that there is an inherent bias toward life as we know it through our own planet's history. The search for life “as we don't know it” then brings this bias forward to decision-making regarding mission instruments and payloads. Understandably, this leads to several top-level scientific, theoretical, and philosophical questions regarding the definition of life and what it means for future life detection missions. How can we decide on how and where to detect known and unknown signs of life with a single biased data point? What features could act as universal biosignatures that support Darwinian evolution in the geological context of nonterrestrial time lines? The purpose of this article is to generate an improved nomenclature for terrestrial features that have mineral/microbial interactions within structures and to confirm which features can only exist from life (*biotic*), features that are modified by biological processes (*biogenic*), features that life does not affect (*abiotic*), and properties that can exist or not regardless of the presence of biology (*abiogenic*). These four categories are critical in understanding and deciphering future returned samples from Mars, signs of potential extinct/ancient and extant life on Mars, and *in situ* analyses from ocean worlds to distinguish and separate what physical structures and chemical patterns are due to life and which are not. Moreover, we discuss hypothetical detection and preservation environments for extant and extinct life, respectively. These proposed environments will take into account independent active and ancient *in situ* detection prospects by using previous planetary exploration studies and discuss the geobiological implications within an astrobiological context.

## 1. Introduction and Motivation

As the discipline of astrobiology is increasing in its parameters and practice for planetary missions, the definitions and usage of terminology that allows for proper differentiation of features from life or modified from life do not yet exist. The broad term of “biosignatures” has been increasingly applied to features on Earth where the burden-of-proof for life is significantly lower than Mars, Europa, and other solar system bodies that evidence past or currently habitable chemistries and liquid water as the solvent that potential life could utilize (Benner, 2010).

Over the last three decades, searching for the oldest preserved signs of life has led to misinterpretations of features preserved in ancient rocks that cannot take into account terrestrial *in situ* “contamination,” in the sense of “younger” biological features inhabiting older features in the rock record (Westall and Cavalazzi, 2011). However, if an independent origin of life did indeed start separately on Mars, a planet without plate tectonics for most of its history, these contamination caveats would be used as supporting arguments for the preservation of ancient organics or perhaps extant life in the subsurface, providing we were able to prove that a positive signature and/or marker were indeed indigenous to the sample and/or site. This is not to say that these efforts are unwarranted, quite the contrary.

However, the robustness of biology on our own planet makes life detection much easier owing to the access of complete laboratory facilities devoted to such analyses and sample handling. The difficulty of life detection and separation of potential “younger” contamination for samples increases in the ancient rock record to degrees where it may not be possible. The significant difference between the burdens-of-proof for the Earth and other solar system bodies requires both independent observations of biogenicity and an agreed-upon nomenclature, encompassing the geological context and the biological feedback.

Clarity for this field is crucial for proper identification of what definitely is created only by biological processes and preserved within rocks and minerals. Many landed robotic planetary missions are focused on signs of ancient or present aqueous activity on planetary bodies or moons (Squyres *et al.*, 2005; Murchie *et al.*, 2009a, 2009b). Mars exploration since Pathfinder in the late 1990s was a proof-of-concept that a rover could indeed land safely on the planet.

It was not until the gamma ray spectrometer and the thermal emission spectrometer (TES) onboard Mars Odyssey detected the Fe-oxide hematite (Fe_2_O_3_) in the plains of Meridiani Planum that the Mars Exploration Rover Opportunity was sent to that site in 2004 (McLennan *et al.*, 2005; McLennan, 2012). It was during the first 6 weeks of that mission that several layered outcrops and associated sulfate eolianites along the rim were observed *in situ* in the Endurance Crater. A short time after Opportunity made this discovery, the Mars Reconnaissance Orbiter Compact Reconnaissance Imaging Spectrometer for Mars (CRISM) started its global campaign to observe planet-wide signs of ancient aqueous environment as potential sites of habitability in late Noachian/early Hesperian waters and the minerals precipitated or modified by *in situ* fluids (Squyres and Knoll, 2005).

**FIG. 1.**
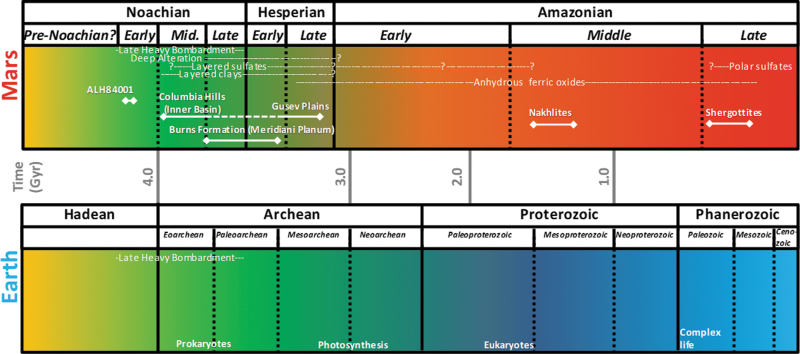
Comparisons of the origins of life on Earth and history of sedimentary features on Mars. Relationship between habitable periods on Earth and Mars. During the periods when the first life evolved on Earth, the martian surface was warmer, wetter, and habitable. The age of sedimentary rocks from the Opportunity rover landing site is ∼3.5 Gyr (late Noachian/early Hesperian). It is also in this time period where the surface of Mars likely had water stable on the surface (McLennan *et al.*, 2005; Squyres *et al.*, 2005) in liquid form versus recent discoveries of surface brine fluids on present-day slopes (Ohja *et al.*, 2015).

How can we constrain habitability on another planet other than Earth without knowing how life would have evolved in a planetary ecosystem? How early in the terrestrial Darwinian evolution should we consider extraterrestrial life even having the ability to adapt to ancient planetary environments (Fig. 1 and Table 1) and their associated terrestrial analogues on Earth? What features of evolution should we consider when assessing life detection? These questions have led the planetary geology community down pathways of directly associating habitability with ancient signs of water on Mars and with potential active subsurface water on Europa.

**Table 1.** The Terrestrial Extreme Environments Where Conditions for Life Are at Its Limit

Parameter limits	Parameters						
	Temperature	pH	Salinity	O2	Desiccation	Radiation	Pressure
Hot (growth >80°C)	Hyperthermophile (*Methanopyrus kandleri*)						
Warmer (growth 60° to 80°C)	Thermophile (*Pyrolobus fumarii)*						
Frozen (growth <15°C, active at - 18°C)	Psychrophile (*Synechococcus lividus*, *Cowellia*)						
Low pH (<5)		Acidophile (*Ferroplasma acidarmanus*)					
High pH (>9)		Alkaliphile (*Alkaliphilus transvaalensis*)					
2 to 5 M NaCl			Halophile (Halobacteriaceae)				
Requires O2				Aerobe			
Tolerates some O2				Microaerophile			
Does not require O2				Anaerobe			
Anhydrobio tic					Xerophile		
Ionizing radiation to 15 kGy						Radiophile	
Pressure-tolerant							Piezophile
							

Examples of extreme environment microorganisms that can potentially survive planetary transit from Earth to Mars, Europa, or other planetary body. Moreover, these metabolisms also have the ability to survive in these solar system environments and would provide useful metagenomic insight into survival strategies for life as we do not know it yet (Adopted from the National Academy of Sciences and Rothschild and Mancinelli (2001).

Life as we know it on Earth can survive in all known climates and extreme ecosystems despite the highest and lowest temperature and pressure endmembers. Life “as we know it” (Table 1) persists in settings that it can adapt to and eventually, over geologic time, can thrive in.

An astrobiology nomenclature needs to consider the availability of the one single data point of life and evolutionary processes that we know of on Earth. This article discusses the shortcomings of life detection and provides a framework for how to define extraterrestrial biology. Moreover, we formulate and discuss the proper definitions for biogenic and abiogenic processes that can lead to better assessments of planetary habitability (Cockell, 2014a, 2014b). This proposed nomenclature will reflect life as we know it and leave enough ambiguity for life as we are yet to discover it.

## 2. Nomenclature and Definition Sets

The definition of life from a textbook will vary depending on the interpretation of the discipline. In biology, if something is alive, then it can respond to its current environment, has a cellular composition, is able to gain energy from chemical reactions (metabolism), has the potential for growth, can replicate itself, can maintain homeostasis, and can inherit properties or traits from previous generations.

Extreme environments that yield high salinity, low a_w_, and radiation tolerance, among others (Table 1), will have specialized gene expression that tolerates the aforementioned extreme properties. Typically, these ecosystems have a lower microbial diversity than nominal/nonextreme settings due to lower adaptability of microorganisms and tolerant gene expressions that would allow the ongoing maintenance of these biological processes (Summons *et al.*, 2008; Msarah *et al.*, 2018; Chaya *et al.*, 2019; Fagorzi *et al.*, 2019; Gugliandolo and Maugeri, 2019).

The current broad nomenclature for detecting these features, however, does not capture temporal and evolutionary features in the general “biosignatures” terminology that many in the planetary science communities use for advocating for mission landing sites and future instrument payloads.

*Chemical Biomarkers* are quantified only by instrumentation and unable to be seen with the naked eye. They are macromolecules from the chemistry of biological processes and interactions with minerals. These biomarkers are usually not arranged in any well-ordered set concerning volume and retention time (Anything else besides volume and retention time?). They are only observable if active biology is present or if the remnants of extinct biology have remained intact since the point of mineral precipitation.

Examples from terrestrial life include nucleic acids (deoxyribonucleic acid, ribonucleic acid), hopanes (Summons *et al.*, 1999), cellulose, lipids (Eigenbrode, 2008; Georgiou and Deamer, 2014) (specifically fatty acids), proteins (specifically polypeptides and repeating monomer units), carotenoids (Perl and Baxter, 2020), among others. It should be noted that the chemistry that forms carotenoid pigments that are visible to the naked eye (in high enough concentrations) are simultaneously chemical in their composition and also presented as physical features.

*Physical Biosignatures* are physically observable formations that can be imaged. Such examples include non-Brownian and independent motion of cellular life (Bedrossian *et al.*, 2017), microfossils (Schopf, 1993), and visible pigments (Fendrihan *et al.*, 2009; Lowenstein *et al.*, 2011; Winters *et al.*, 2013; Perl and Baxter, 2020). It should be noted that pigment components are both a visible feature and have a chemistry that would be a biomarker, and so, these satisfy both categories simultaneously (Perl and Baxter, 2020). These visible features can be seen with the naked eye, μ-scale to mm-scale images, and do not require instrumentation other than images or video. For extinct biology, these physical signs of life can be evident if the preservation medium has been maintained and if no physical or chemical modification occurred after the last instance of biology or a biological process took place. For extant life, these features should be able to respond to forms of chemotaxis, phototaxis, or pigment generation.

In order for the evidence of life as we know it to be significant, both *chemical biomarkers* and *physical biosignatures* would need to be independently measured in multiple parts of a sample. This is taking into account extinct (ancient) life where the preservation medium plays a significant role and extant (active) life where availability of sample is less of an abundance concern. Evidence of ancient life relies heavier on the preservation medium for any chemical biomarker and physical biosignature, whereas chemistry and physical features of active life could be readily available.

These two overarching definitions need widely different lines of evidence before a burden of proof is established. If either the *chemical biomarker* or the *physical biosignature* in question in an unknown sample can be established, and not both, the evidence will likely fall short for the burden of proof needed for astrobiology and a second sign of life in our solar system. Should these physical and chemical features co-occur and remain preserved over geologic time, then the interpretation, with respect to their visual/physical and chemical analysis, should fall into the following four categories:

*Biotic*: a feature or measurement that would only exist if biology generated it or it was undoubtedly modified by life. Without the processes from life, this measurement or feature would not exist. Notable examples of this would include nucleic acids RNA and DNA.

*Biogenic*: a feature or measurement that is found with relationships to biological processes but may exist (or be consumed by) without the influence of life. This feature would look different depending on the type of biological processes involved.

The difference between biotic and biogenic would be a measurement of life directly and a measurement of a process generated by life, respectively. An example of biotic would be the visual and chemical measurement of bacteria, while an example of biogenic would the calcium carbonate (CaCO_3_) precipitated onto bacterial extracellular polymers (Tourney and Ngwenya, 2009) by life.

*Abiogenic*: a feature or measurement that is found equivalently with and without associations to biological processes with its existence not contributing to any biomarker or biosignature. This is often difficult to quantify for Earth due to the abundance of extant life and what is preserved in the rock record.

*Abiotic*: a feature or measurement that has no relationship to life at all and whose existence would be visually and/or chemically interchangeable with or without the presence of biology or biological processes and has undeniably no relationship with biology, past or present.

An example of an abiogenic measurement would be the presence of methane (CH_4_) in a measurable source without associations to biology, such as when these natural gases are generated and trapped in Earth's mantle (Scott *et al.*, 2004). A positive CH4 measurement does not conclude that its sources are from life's processes, only that it is present. However, an abiogenic measurement can become biogenic depending on the relationships between positive detections and their biological sources. With regard to the aforementioned methane example, should the carbon source of the methane be higher in the lighter ^12^C isotope and alongside formaldehyde, methanol, and other trace gases, then these would have a higher likelihood of having a biological origin.

Finally, should these measurements have an energetic flux [*i.e.*, increasing volumes due to biological activity during daytime because of cellular energy consumption via photosynthesis (Westall *et al.*, 2011; Bansal *et al.*, 2018)], then it would further add to responding to a high burden of proof-for-life validation (Kiang *et al.*, 2007).

An example of an abiotic component can be treated spatially, chemically, or temporally. An example of metal-reducing bacteria can be used to illustrate all three. Fe-reducing organisms are able to utilize Fe(III), Mn(IV), or other electron acceptors depending on the abundance and proximity. In the case of these terminal electron acceptors being part of the rock record, the host rock itself could contain Fe(III) and would be utilized by the iron reducers.

The parent rock itself has no impact on this electron transport chain, but its spatial position in the rock record is necessary to compartmentalize the energy source. Chemically, as long as the parent rock is stable (and potentially helps preserve the electron acceptors), it does not provide any redox reaction to the nutrient chain. Consequently, if the parent rock is not stable over time and does fracture, the addition of younger material to this hypothesized system, should it not influence any of the aforementioned processes and/or occurs after extant life has perished, would be abiotic due to not overlapping with any of the electron transport chains.

Ironically, the further away from biotic you perceive these examples to be the more difficult it is to describe. If features on Earth are indeed from life, it is quite simple to make *in situ* measurements and study the operational taxonomic units and metabolomics of a system (Seyler *et al.*, 2020). As we live on a microbially diverse planet teeming with life, the search for uninhabited regions still within the thermodynamic, pressure, and temperature endmembers for supporting metabolic processes is a more difficult endeavor.

**FIG. 2.**
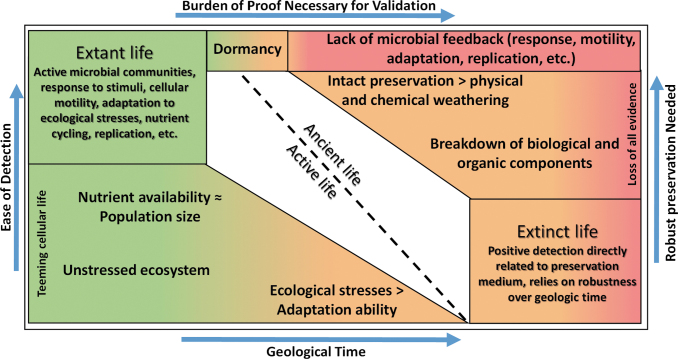
Conceptual probabilities of validation of extinct and extant life. Astrobiological mission strategies need to move toward life validation alongside life detection. For extinct (ancient) life, the probability of detection of former biological components or other true biological features are at the mercy of the preservation medium and its robustness over geologic time. For extant (active) life, the availability of “positive” detections is only limited by the instrumentation and physical proximity to the microbial communities.

These categories can be heavily dependent on the terrestrial *in situ* setting that life and its habitats would utilize for nutrient cycling (*i.e.*, the Fe mineralogy used by Fe-reducing bacteria (Luef *et al.*, 2013), elemental carbon or sulfur in fluid inclusions for entombed halobacteria, being some examples, Mancinelli *et al.* 2004; Perl and Baxter, 2020). For planetary exploration on Mars into outcrops and features laid down by flowing ancient waters, these features could have contributed to extant life on the planet (if it existed in the first place), but by themselves do not provide enough evidence to establish it is biotic (Fig. 2).

## 3. Probability of Life Detections: Moving Toward Life Validation

The focus of the Mars program at the beginning of the *Spirit* and *Opportunity* rover missions had been to “follow the water.” The discovery of layered bedrock, the presence of spherical Fe-oxide concretions (“blueberries”), and a host of other water/rock features at Meridiani Planum showed that this site was host to several groundwater recharge events (McLennan *et al.*, 2005; McLennan, 2012) and had a stagnant paleo-groundwater table for a significant amount of time (Ehlmann *et al.*, 2011). Later with the *Curiosity* rover mission to Gale crater (Hurowitz *et al.*, 2017) and the upcoming *Perseverance* rover mission to Jezero crater (Lapôtre and Ielpi, 2020), the shift to habitable environments on Mars has linked the ancient stable surface waters of the late Noachian to areas where sedimentary outcrop could reveal signs of life (Westall *et al.*, 2015).

Should an independent origin of life have occurred on Mars, and if it utilized these stable surface and subsurface waters as its solvent for metabolic processes, these sites would have the highest probability of detection due to direct water/mineral interactions with the potential to be preserved in a layered sedimentary deposit (Cockell, 2014a). Up until recently, the focus for Mars mission objectives was the search for extinct life and how it could be preserved (Summons *et al.*, 2014). Carrier *et al.* (2020) noted a new interest in extant life on Mars with sites and features that could harbor active microbial communities.

For Europa, Titan, and Enceladus, the focus is on extant life due to active solvents on these moons (water, liquid ethane, and methane) (Cable *et al.*, 2012) buried beneath kilometers of ice. Moreover, Enceladus is being looked at for a prebiotic potential (Kahana *et al.*, 2019). The flyby missions of Galileo and Cassini have shown active liquid plumes erupting from the surface and observations by the Hubble Space Telescope have even captured these features from the Earth (Sparks *et al.*, 2017).

Later this decade, part of the Europa Clipper mission (Howell and Pappalardo, 2020) will hopefully quantify and observe these features with future landed science payloads targeting ways to dig beneath the ice layers to get to potentially active microbial life in the subsurface ocean (Priscu *et al.*, 1999). Current mission concepts and studies include ESA's Jupiter Icy Moon Explorer, the Europa Lander concept, NASA's Scientific Exploration Subsurface Access Mechanism for Europa, and the Honeybee Robotics Search for Life Using Submersible Heated Drill, among others.

The common habitable environments and life detection payloads of both extinct and extant life mission objectives tend to yield overlapping elements. Some sort of visual imager or camera [Pancam, MastCam, MastCamZ, Europa Imaging System (Squyres *et al.*, 2005; Wellington *et al.*, 2017; Centurelli *et al.*, 2018); a chemical analyzer for elemental and mineralogical analyses (VNIR and IR spectrometers (CRISM) (Murchie *et al.*, 2009a, 2009b; Viviano-Beck *et al.*, 2014))], laser-induced mass spectrometer (CheMin) (Blake *et al.*, 2012), alpha particle X-ray spectrometer (Rieder *et al.*, 2003), Mini-TES (Christensen *et al.*, 2003), Europa-UVS, Mapping Imaging Spectrometer for Europa (Retherford *et al.*, 2015; Bender *et al.*, 2019); a gas chromatography suite [Sample Analysis at Mars (Eigenbrode *et al.*, 2018), Mass SPectrometer for Planetary EXploration/Europa (Brockwell *et al.*, 2016); and a radar system (Radar for Europa Assessment and Sounding: Ocean to Near-surface (Pappalardo *et al.*, 2013), Mars SHAllow RADar sounder (Nunes *et al.*, 2011))]. On Earth these instruments can be used to document distinct features of life after we have already confirmed where it has thrived or impacted the mineral and rock records.

The measurements of *in situ* organic compounds on both Mars (Eigenbrode *et al.*, 2018) and the ocean worlds (Waite *et al.*, 2017; Kaplan *et al.*, 2018) are steps in the right direction for potential biological detection with future mission payloads, but how would we design a mission and its architecture to validate life as we do not know it? Would it benefit us to send a DNA extraction system to a martian recurring slope lineae site or a plume eruption from Europa? If we intend to prove that life as we do not know it did not come from terrestrial evolution, but still has properties of Darwinian evolution, it may behoove us to focus on the utility of biological compounds (Lovelock, 1965). Their function on Earth and through terrestrial geological time could still apply (Kish and DiRuggiero, 2012).

The evolutionary need for transfer of genetic information and surviving gene expressions, the ability to replicate, reactions to stimuli, adaption to ecological stresses (Jones and Baxter, 2016), maintenance of homeostasis, and organization of cellular compartments—these are all characteristics of life as we know it and potentially could be used as universal biomarkers (chemical) and biosignatures (physical) due to their measurable presence and independent of being locked into issues of contamination versus *in situ* signal. Being able to incorporate these utility-driven features into future instrument payloads sidesteps the terrestrial bias of life as we know it. If an independent tree-of-life started elsewhere in our solar system and life thrived there, the aforementioned traits would need to exist in parallel with each other (Fig. 5).

### 3.1. Extinct (ancient) life vs. extant (active) life

**FIG. 3.**
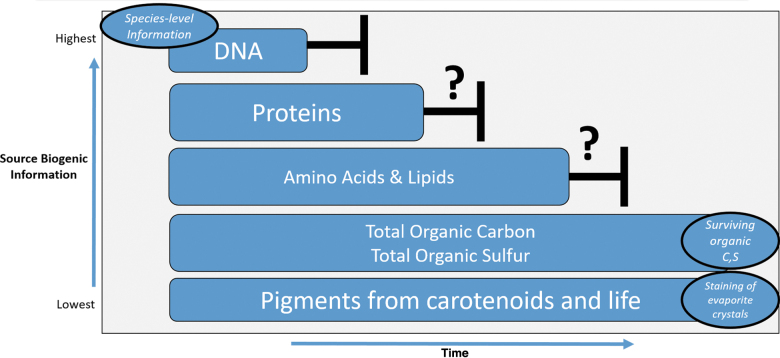
Biogenic preservation and biotic information over modern and geologic time. High-level investigation space using terrestrial life (“as we know it”) and the loss of biotic information within biogenic preserved settings with respect to time.

The question sets of validating extinct life and extant life do overlap in content but differ in volume, molecular complexity, and spatial distribution (Marshall *et al.*, 2017). Contamination concerns notwithstanding, a positive detection of extinct life in the form of a robust lipid, layered stromatolite, or other true biological features are at the mercy of the preservation medium (Fig. 3).

Summons *et al.* (2014) discussed the probability of lacustrine environments (Lynch *et al.*, 2015) and its features preserving signs of life for the planned Mars sample return campaigns. These features on Mars would have had their “best” preservation abilities when water was abundant and stable and the desiccated surface was not exposed to UVC radiation that allows for cell lysis. There are physical considerations where even within such a hostile setting, specific minerals and sedimentary outcrop features can protect against such detrimental processes (Perl *et al.*, 2019). Aside from the natural degradation of sensitive nucleic acids and other more robust biological compounds, these features need to continually be examined for preservation metrics over geologic time since they would allow for the best defense against irradiation and desiccation.

If we assume that extant life in a martian or europan environment follows Darwinian evolution and it is in a “steady-state” form (implying that it has adapted to the majority of ecological stresses that have naturally occurred in its environment), then any extant biology inhabiting an environment would have an abundant presence only constrained by stresses too hostile for their maintenance of metabolic processes and homeostasis (Fig. 2). Preservation metrics in the extant life case could isolate some microbial communities and create population differences with a common ancestor occurring at the temporal point before separation (Hedge and Wilson, 2016) but would not be a direct influencer of a potential biological measurement due to the ongoing microbial activity.

### 3.2. Use case: preservation of nucleic acids and carotenoids in evaporites

Features from mineral/microbe interactions in modern and former hypersaline settings can simultaneously act as both a marker for aqueous environments and signs of halophilic extant or extinct life. Evaporite minerals can capture and entomb organic matter within their intercrystalline and intracrystalline structure as inclusions because they precipitate relatively quickly (nomenclature adopted from Schopf *et al.*, 2012 and in further detail in Perl *et al.*, 2020) and can further preserve metabolic processes within the fluidic structures. Thus, evaporite minerals constitute a target for biosignature investigation on Earth and Mars, where evaporitic deposits exist. However, little is known about the process of organic preservation and detection limits in evaporites, or the stability of such molecules when exposed to significant UV radiation (as would be present on the surface of Mars).

Previous results (*e.g.*, Vítek *et al.* 2009; Jehlička and Oren, 2013; Winters *et al.*, 2013; Jehlička *et al.*, 2014; Perl and Baxter, 2020; Perl *et al.*, 2020) show that β-carotene has a strong Raman signature that remains strong even when entombed in halite. One study shows that the β-carotene trapped in fluid inclusions and subjected to intense UVC radiation changed little even after several weeks, while β-carotene not trapped in halite degraded quickly. The authors' (Perl and Baxter, 2020) results reveal that complex organic molecules such as β-carotene should be preserved well in halite, especially in fluid inclusions, and that halite does provide some protection from organic matter degradation from UVC radiation.

**FIG. 4.**
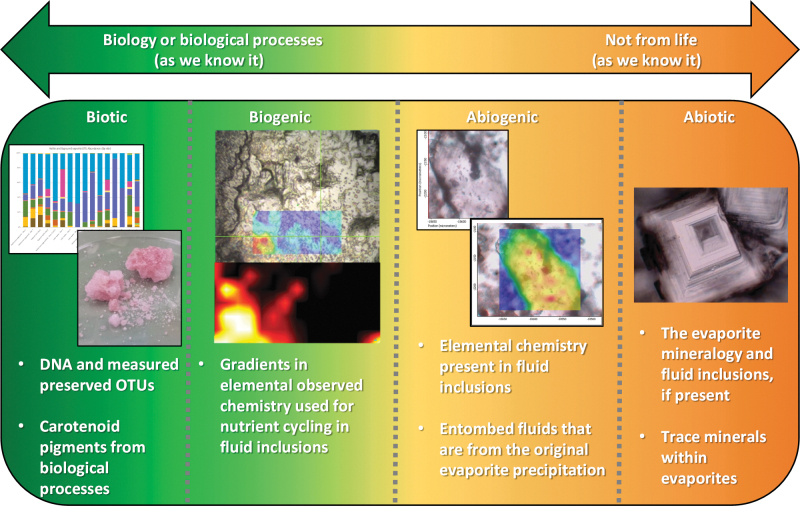
“Pendulum” diagram showing examples and definitions of the proposed astrobiology nomenclature for an NaCl hopper crystal. The example above is for a single pigmented halite hopper crystal (biogenic) and then brought from left-to-right showing how features lose their biological validity due to the definitions proposed in this aricle (Original credit: Frank A. Corsetti).

Isolated pockets of brine trapped in halite crystalline structures have been used to study the ancient environments of where fluids originated as well as microorganisms from ancient waters (Satterfield *et al.*, 2005; Benison *et al.*, 2008; Lowenstein *et al.*, 2011). Thus, evaporites constitute a good target for the search for biomarkers on Mars. These findings will allow for proper criteria (Fig. 4) for the discovery of any potential physical biosignature and chemical biomarker that would be on active ocean worlds (Europa, Enceladus) and for future Mars subsurface roving or drilling missions (Lunine *et al.*, 2015).

### 3.3. Use case: differences in total organic species from biological and nonbiological sources

Many planetary missions, including Cassini and the Mars Science Laboratory, have been or are equipped with the ability to detect organic species. Organic molecules are necessary for life; however, it is well accepted that there are multiple abiotic routes to the synthesis of organic compounds such as amino acids and nucleotides (McDonald and Storrie-Lombardi, 2006), and even of more complex molecules such as oligomers. The combination of biotic and abiotic synthesis routes makes the use of individual organic compounds as biosignatures challenging. Some researchers have considered the ratio of different biologically relevant compounds to be a biosignature, able to distinguish between the biotic and abiotic syntheses of the organics (McKay, 2004, 2011); however, changing environmental parameters can also affect organic distribution patterns in abiotic systems (Barge *et al.*, 2019).

More laboratory-based experiments would help elucidate the ability of abiotic chemistry under diverse geological settings (Georgiou, 2018). Better understanding of the abiotic ratios of organic molecules will limit the number of false positives observed on planetary science missions especially as we continue to explore worlds that we believe could be habitable.

### 3.4. Caveats and false positives

The burden of proof for life (as we do not know it) is compounded by the methods we seek to detect and validate such evidence. If we had the capability of terrestrial research laboratories on Mars, Europa, and the other ocean worlds, then multiple experiments and limitless sample return opportunities would be present. Budgetary, sample mass, rover power source, rover size, flight time, and a whole host of other technical issues will always prevent this until planetary exploration missions have the capability to have sustained human presence on these astrobiologically relevant bodies. Until then, we will rely on robotic missions, remote observations, and *in situ* analyses of regolith, mineral, and liquid samples.

One of the recommendations from the National Academy of Sciences Astrobiology Strategy (National Academies of Sciences, Engineering, and Medicine, 2019) for the Exploration of Mars was “selection of samples for analysis (either *in situ* or samples returned from Mars to Earth, McCubbin *et al.*, 2017) should emphasize those having the best chance of retaining biosignatures.” This decision process must consider eliminating false positives from the beginning of *in situ* sample collection onward to when these samples are returned to Earth for laboratory study.

Given the ambiguity of results from martian meteorite ALH 84001 (McKay, 1997; Thomas-Keprta *et al.*, 1998), one key lesson learned was that morphology alone is not enough to determine biogenicity (García Ruiz *et al.*, 2002). While this may be self-evident at present, this fact can be used as an argument for sample collection based on unique empirical observations on the mm- or meter-scale in martian (*e.g.*, stromatolite-type features, evaporite mineral pigments) or europan/enceladen ice features (*e.g.*, ambiguous cell motility, unique/patterned ice layering). Distinguishing between false positives in terrestrial samples (Cady *et al.*, 2003; Cady and Noffke, 2009; Emerson *et al.*, 2017; Reinhard *et al.*, 2017; Harman and Domagal-Goldman, 2018; Neveu *et al.*, 2018; Cockell *et al.*, 2019) continues to be necessary for planetary analogues.

Should returned samples from Mars yield no positive sign of life, there would be several distinct possibilities for this (Cockell and McMahon, 2019) and would not be representative of the entire planet. Some of these prospects include if the sample collected from Mars did not originate on Mars itself, if biosignatures within the sample did not originate from within the sample and endure to present-day discovery, if the signs of life cannot be separated from its abiogenic and abiotic components, or if the volume of the preserved life cannot be detected.

## 4. Validation of Life and Removing the Terrestrial Biases of Life Detection

While the official statement from Lourens Baas Becking may have been lost to time, he is loosely quoted (Wit and Bouvier, 2006) as saying for life, “*Everything is everywhere, but the environment selects.*” While this was largely meant for terrestrial biology, the application of this to our solar system and the Universe provides an interesting paradox for life detection and our perspectives for searching for life as we do not know it. Terrestrial microbial evolution at present is not a sufficient metric for life elsewhere, due to likely evolutionary differences between Earth's geologic and climate histories and other habitable solar system bodies. Stated differently, we should not focus our search on macromolecules (such as DNA) outside of Earth. DNA is a product of Earth evolution and the eventual output of the original Last Universal Common Ancestor (LUCA) on our own world.

Rather than DNA alone, the utility of nucleic acid oligomers as an information transfer component should be considered. Within the search for life in our solar system, the ability to search for a “DNA-like” compound that fulfills the transfer of genetic information between generations could fulfill both the metric for validation of an independent biology and being able to place it in a second tree-of-life. The more specific and closer to our present-day evolutionary markers we get, the more distant we would be from a separate evolutionary pathway or separate tree-of-life whose evolution would be different both from the standpoint of a non-Earth LUCA and from different temporal, biogeochemical, and planet-wide trajectories (Scharf *et al.*, 2015; Hug *et al.*, 2016).

In short, life has evolved on Earth due to the geological, chemical, and environmental histories that our planet experienced. During these events, early cellular life as we know it evolved and responded to changes to our planet over time and led to the genetic makeups and ecological systems as we know them today. To try to search for those same systems outside of Earth makes the incorrect assumption that other planets and moons shared the same LUCA, the same planetary evolution, and the same microbial response over geologic time.

## 5. Discussion

The hybrid nature of geobiology has allowed for interpretations into mineral/microbial interactions from the perspectives of terrestrial microbial ecology and evolution to be studied as a reference for life as we know it. Until now the lack of a utilized nomenclature has led many abiotic and abiogenic features to be misclassified as potentially modified from life, younger contaminated features into ancient mineralogy (Vreeland *et al.*, 2000), or unitless measurements of habitability that do not take into account evolution and adaptation (Ehrenfreund *et al.*, 2011). These previous studies are critical for understanding and constraining biogenicity in fluid-precipitated (evaporite) samples and can be used as baseline constraints for future Mars Sample Return studies.

On Earth and other habitable solar system bodies that could harbor life, it would be valid to state that biology, acting faster than geology, can adapt to planetary changes that would occur over geologic time (Fig. 1). Should those changes continue to propagate over geologic periods and globally those differences in habitability not change too significantly, life as we do not know it should be observable on a larger scale (Seager *et al.*, 2005) than the approach that planetary missions have taken in the last five decades.

The need for nomenclature to study astrobiological features potentially generated or modified by non-Earth biological processes is paramount for the proper interpretation needed for future data analysis of unknown samples that are planned to be returned from Mars or ocean worlds within the next decades. While no concrete plans have been set for how to return samples from Mars and how (and which) laboratory analyses on Earth will be conducted within a modified BSL environment, significant thought to preventing contamination needs to be undertaken.

Following the first “stage” of sample collection by the *Perseverance* rover, samples will likely be cached and left on the martian surface. Should any organic components be preserved in these sedimentary deposits, the quantitative yield between *in situ* organics would be very sensitive to any modern contamination. Moreover, should any less robust biological components be present (extant life), the risk of contamination and the inability to decipher between terrestrial contamination and that biology are significantly higher.

It is still unknown how these samples would be analyzed if they can make it back to Earth intact and what types of geobiological and microbiological laboratory work would be conducted to determine whether life ever was present on Mars. However, the types of analyses that should be done would be very similar to what we currently do, and these analyses may not yield the results that we would expect. Our modern-day laboratories and analytical strategies are geared toward life as we know it.

Before any Earth analyses on martian samples, these soil (and hopefully evaporite mineral) samples should be inspected nondestructively and visually for any physical biosignatures that may have modified the mineral or sediment. Only after visual inspection and μ-scale assessment have been made should destructive chemical biomarker analysis take place. This can include liquid chromatography for any lipid preservation and potential metagenomics to see whether we have contaminated the samples with any terrestrial by-products of the initial sample capture on Mars.

Would nonterrestrial life have the same common elemental chemistry as we know of on Earth? Is something as simple as organic carbon in a unique looking microstructure to prove that a feature is indeed biogenic and not just organic? If we go back to the Baas Becking hypothesis, we should conclude that we would not have to look very hard if we had a hand sample from another planet that was teeming with life. We should see life's unique properties all over the sample, much like algae atop a pond or worms underneath a rock. Does this mean that since we see nothing alive on the surface of Mars that there is no life there now, or nothing biological was ever present?

The lack of evidence on Mars's surface does not infer anything for the shallow subsurface or even deeper subsurface regions, kilometers below the martian crust. We do know that groundwater at pH ranging from ∼2 to 4 to near-neutral levels in Meridiani Planum (Knoll *et al.*, 2005; Tosca *et al.*, 2005; Filiberto and Schwenzer, 2018) and Gale Crater (Meslin *et al.*, 2013; Rapin *et al.*, 2019), respectively, had the ability and vertical range to make way through permeable sedimentary rock and breach the crust. We also know that some of these fluids' sources were closed basin lake systems, where salinity was likely 10-fold higher than Earth marine waters are now.

If life was ever present on Mars and it resided in these closed basin systems, then the eventual downward movement of these ancient waters after the loss of the martian atmosphere would have provided a haven from the UVC and global desiccation that would eventually occur over the next ∼3.5 Gyr into the Amazonian (Michalski *et al.*, 2013). Given the duration of this groundwater, downwelling waters still exposed to the surface would have eventually frozen.

**FIG. 5.**
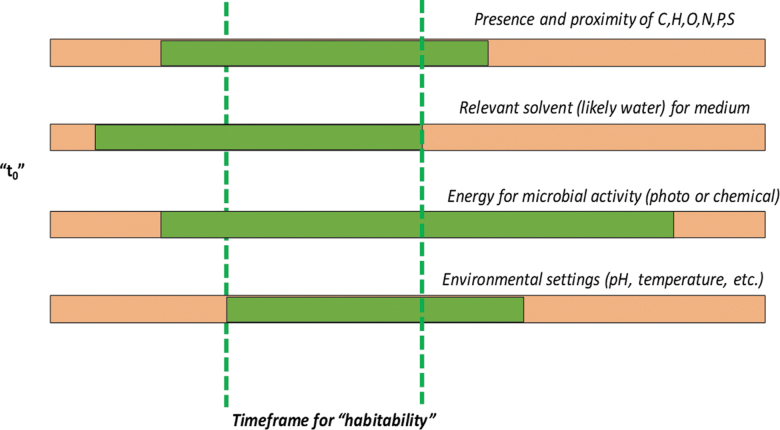
A proposed update to the classical perspective of habitability for planetary systems. The classic Venn diagram of energy sources, solvents, climate conditions, and C, H, O, N, P, and S has been used for discussions into martian habitability and taking global and local measurements from orbiters, rovers, and landers to determine how “habitable” a location was. That assessment did not take into account the need for the four environmental and aqueous features to overlap in time. It would only be the time frame of the combined overlap of all four products that life, as we know it, would have the highest probability for survival after a separate origin and last universal common ancestor should microbial evolution took place outside of Earth. Box 2.2 of the National Academy of Sciences Dynamic Habitability chapter in *An Astrobiology Strategy for the Search for Life in the Universe* discusses these features in further detail.

This aqueous downwelling and likely hypersaline values in these waters would have led to significant precipitation of subcrustal evaporite layers where the water became stagnant and even deeper still. If cellular life utilized the current categories of habitability and overlapped each other (Fig. 5), that would yield a high probability that preservation could have occurred.

## 6. Conclusion and Pathways Forward

The best way forward for astrobiology is to integrate planetary geology with terrestrial microbiology so that the communities understand how each discipline formulates the research questions for the joint *in situ* sample analysis and continued global observations of Mars. Should we ever find something *in situ* that fulfills the requirements both for a physical biosignature and a chemical biomarker, the next question will involve identification and classification.

In turn, this will lead to noteworthy questions involving martian cellular life, metabolisms in the deep subsurface of Mars, and evolution outside of Earth. For the ocean worlds and the prospect of extant life, the burden of proof needed is not limited by a preservation medium. Moreover, the volumes of extant biological samples would likely be magnitudes higher than in a preserved state after life has died out. However, if life were ever present on a rocky planet that became inhabitable over geologic time, the ability for biology to evolve and adapt strategies for survival in subsurface ecosystems (in the case for Mars) could have been utilized by life via permeable sedimentary rocks and groundwater downwelling into shallow subsurface aquifers.

The burden of proof needed for validating a second sign of life in our solar system is significantly higher for ancient/extinct life than active/extant life (Fig. 2). Focusing our efforts toward what Darwinian evolution would yield over geologic time allows our science mission objectives to be designed with validating life rather than limited to life detection, which can inherently contain biases from terrestrial biology. Robotic missions, which are inherently limited with respect to laboratory capabilities due to power and mass constraints, compound these issues.

Should an independent origin of life have occurred on Mars ∼3.5 Gyr, the genomic expressions that would have been necessary for survival would have had to include the halotolerance as well as low a_w_ settings, while still maintaining metabolic processes. Photobiological feedback in the form of pigments would be an ideal survival strategy for halophilic microorganisms as the planet became more irradiated with UVC (Litchfield, 1998; Perl and Baxter, 2020). As the focus for Mars starts to include extant life studies, the late Noachian preservation of these features is of paramount importance (Carrier *et al.*, 2020).

For the icy moons where present-day subsurface oceans may exist (Pappalardo *et al.*, 2013), we would consider cellular motility (Bedrossian *et al.*, 2017) and the presence of complex molecules that could be the framework for life as we do not know it. If we assume some form of Darwinian evolution to be the same for nonterrestrial life, then information transmission between growing microbial communities would allow the adaptation of cellular life in these subglacial oceans.

Strategies would then need to focus on traversing beneath the ice for ocean world exploration and *in situ* sample analyses. Similar strategies could be used for rocky planet subsurface exploration on Mars where many of the halotolerant survival strategies could be utilized far away from surface UVC irradiation.

## Abbreviations Used

CRISM: Compact Reconnaissance Imaging Spectrometer for Mars
LUCA: Last Universal Common Ancestor
TES: thermal emission spectrometer
